# Effects of Postprocess Hot Isostatic Pressing Treatments on the Mechanical Performance of EBM Fabricated TI-6Al-2Sn-4Zr-2Mo

**DOI:** 10.3390/ma13112604

**Published:** 2020-06-07

**Authors:** Miguel Lopez, Christina Pickett, Edel Arrieta, Lawrence E. Murr, Ryan B. Wicker, Magnus Ahlfors, Donald Godfrey, Francisco Medina

**Affiliations:** 1 Department of Mechanical Engineering, The University of Texas at El Paso, El Paso, TX 79968, USA; egarrieta@utep.edu (E.A.); rwicker@utep.edu (R.B.W.); frmedina@utep.edu (F.M.); 2 W.M. Keck Center for 3D Innovation, The University of Texas at El Paso, El Paso, TX 79968, USA; cdpickett@miners.utep.edu (C.P.); lemurr@utep.edu (L.E.M.); 3 Department of Metallurgical, Materials and Biomedical Engineering, The University of Texas at El Paso, El Paso, TX 79968, USA; 4 Quintus Technologies LLC, Lewis Center, OH 43035, USA; magnus.ahlfors@quintusteam.com; 5 Honeywell Aerospace, Phoenix, AZ 85034, USA; donald.godfrey@honeywell.com

**Keywords:** electron beam melting, Ti6242 alloy, post-process HIP, microstructures, mechanical properties

## Abstract

An essentially fully acicular alpha-prime martensite within an equiaxed grain structure was produced in an Electron Beam Melting (EBM)-fabricated Ti-6Al-2Sn-4Zr-2Mo (Ti6242) alloy using two different Arcam EBM machines: An A2X system employing tungsten filament thermionic electron emission, and a Q20 system employing LaB6 thermionic electron emission. Post-process Hot Isostatic Pressing (HIP) treatment for 2 h at 850, 950, and 1050 °C resulted in grain refinement and equiaxed grain growth along with alpha-prime martensite decomposition to form an intragranular mixture of acicular martensite and alpha at 850 °C, and acicular alpha phase at 950 and 150 °C, often exhibiting a Widmanstätten (basketweave) structure. The corresponding tensile yield stress and ultimate tensile strength (UTS) associated with the grain growth and acicular alpha evolution decreased from ~1 and ~1.1 GPa, respectively, for the as-fabricated Ti6242 alloy to ~0.8 and 0.9 GPa, respectively, for HIP at 1050 °C. The optimum elongation of ~15–16% occurred for HIP at 850 °C; for both EBM systems. Because of the interactive role played by equiaxed grain growth and the intragrain, acicular alpha microstructures, the hardness varied only by ~7% between 41 and 38 HRC.

## 1. Introduction

Since the inception of laser and electron beam powder bed fusion processing, Selective Laser Melting (SLM) and Electron Beam Melting (EBM), a major focus of alloy component fabrication has been Ti-6Al-4V, as a consequence of its wide range of applications, particularly aerospace and biomedical [1,2,3]. Unlike more conventional Ti-6Al-4V (Ti64) cast and wrought processing, SLM and EBM processing of Ti64 often produces variances in microstructure and corresponding mechanical properties which may or may not be acceptable in a specific engineering application. For example, SLM involves limited powder bed heating which can result in rapid component cooling. This can produce a preponderance of either a fine, acicular alpha phase originating in the prior beta grains, or a correspondingly fine alpha-prime martensite along with residual stress accumulation, which, in addition to some porosity, can be eliminated by post-process Hot Isostatic Pressing (HIP) treatment. EBM, on the other hand, generally eliminates any residual stress as a consequence of higher-temperature powder bed heating, and the formation of larger, lenticular alpha phase grains in the prior beta grains. Process parameter manipulation can allow for rapid cooling to produce variations in acicular alpha grains or martensite (alpha-prime) [4]. The corresponding mechanical properties can involve tensile yield stress and ultimate tensile strength (UTS) values ranging from >1 GPa, with elongations (ductility) of 7–8% in contrast to tensile yield stress and UTS values ranging from 0.85 to 0.95 GPa, and elongations of >10% for cast and wrought Ti64 products [5,6,7,8]. In this regard, there have been numerous, recent studies involving post-process SLM and EBM heat treatment schedules which, as a consequence of the Ti64 applications, have produced more uniform and stress-free microstructures with superior mechanical properties; in particular, the raising of the elongation to >15%, nearly double the ductility of the as-fabricated products [9,10,11,12,13,14,15,16,17,18,19].

While Ti64 has been one of the most prominent titanium alloy compositions, there have been a host of other Ti-alloy developments over the past half-century [1,2,6,7]. One such alloy has been Ti-6Al-2Sn-4Zr-2Mo (Ti6242), which, in contrast to the alpha/beta Ti64, is a near alpha alloy with mechanical properties roughly 10% greater than Ti64. With a melting point of 1580 to 1716 °C (in contrast to 1604 to 1680 °C for Ti64), Ti6242 has been successful in providing creep resistance up to operating temperatures of 550 C in applications where this is a critical issue. Although there have been studies of Ti6242 in welding applications, including microstructure studies showing the evolution of acicular alpha and fine alpha-prime [20,21], there are very few studies involving the SLM or EBM fabrication of Ti6242, or related post-process heat treatments to optimize both the microstructure and the mechanical behavior [22]. Cai, et al. [23] have also recently reported on the SLM and heat treatment of a Ti-6Al-2Zr-1Mo-1V near alpha alloy as well. It is also worth noting that a recent study of friction-stir processing of Ti6242 by Dutt, et al. [24] showed that the creation of an alpha-double-prime nanoplatelet structure produced a yield stress of ~2 GPa, nearly 70% greater than that of wrought Ti6242; with a corresponding elongation of ~5%.

The present investigations report for the first time the EBM fabrication of Ti6242, and preliminary post-process HIP heat treatment at various temperatures. This study includes the characterization of as-fabricated Ti6242 microstructures and associated mechanical properties (tensile yield stress, UTS, elongation, and hardness) along with comparisons of HIP-related microstructures and the corresponding mechanical properties. The results for two separate EBM systems were compared and found to be essentially the same.

## 2. Materials and Methods

### 2.1. Powder Feedstocks

**Figure 1:**
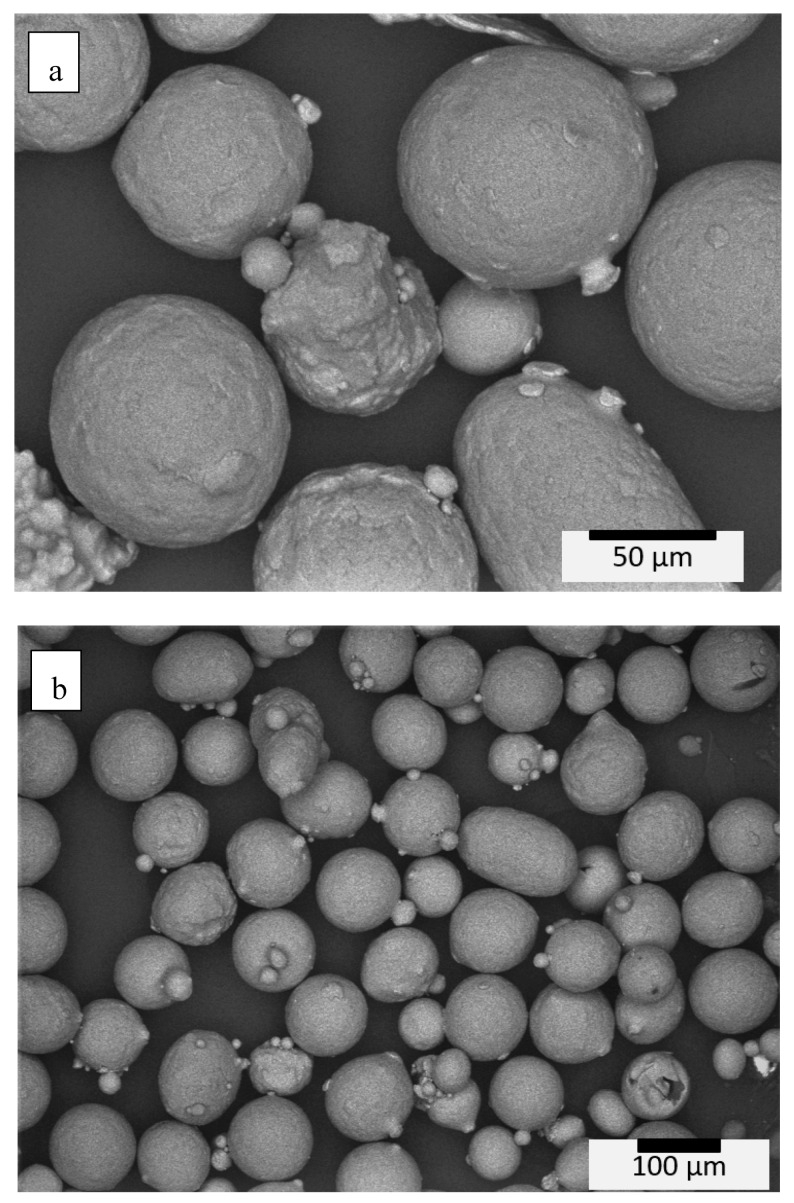
Ti6242 pre-alloyed powder images at high magnification (**a**) and low magnification (**b**).

The Ti 6Al-2Sn-4Zr-2Mo precursor powder used for EBM fabrication was obtained from Carpenter (Carpenter Technology, Camarillo, CA, USA). The powder was produced through gas atomization. The received powder used for E-PBF (EBM) processing was analyzed using a RETSCH-CAMSIZER X2 (Retsch GmbH, Haan, Germany). Images taken of the powder (Figure 1) revealed that a range of particle sizes from ~40 to 95 µm was obtained, with an average of ~71 μm.

### 2.2. Electron Beam Powder Bed Fusion Systems, Setup and Fabrication

Arcam A2X. This machine offers a building envelope of 200 × 200 × 380 mm. It was designed for the production of functional parts within the aerospace and general industry, featuring the capability to withstand high temperatures, up to 1100 °C, with a maximum beam power of 3 kW, using a tungsten filament as its cathode.

Arcam Q20. This machine offers a building envelope of 350 × 350 × 180 mm. It was designed for the production of aerospace components such as turbine blades and airframe components. This system also features a maximum beam power of 3 kW, using a LaB6 cathode.

### 2.3. Process Parameters

Table 1 and Table 2 list the complete set of fabrication parameters for the Arcam A2X and Q20 EBM machines, respectively. The melt parameters—power, beam scan speed, beam dimensions, and layer thicknesses—were essentially the same for the two EBM systems, producing similar, high cooling rates for the fabricated products. These parameters are listed in Table 1 and Table 2. In addition, the tables list parameters specific to the machine building programs, and are listed to enable the specific build parameters used in this preliminary study to be reproduced.

### 2.4. Hot Isostatic Pressing (HIP) Parameters for Post-Processing Heat Treatment

**Figure 2:**
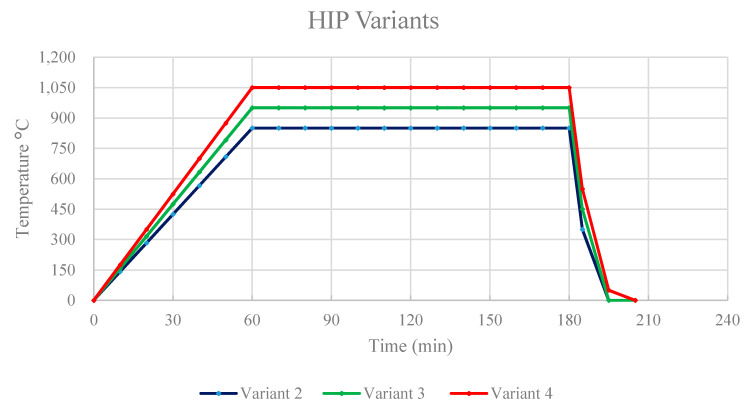
Comparison of different Hot Isostatic Pressing (HIP) cycles for Ti6242 specimens.

The HIP process applies high pressure to the exterior of a part via an inert gas. The elevated temperature and pressure cause sub-surface voids to be eliminated through a combination of plastic flow and atom/vacancy diffusion. In this project, the specimens were exposed to several Hot Isostatic Pressing parameters, separating into different groups according to different HIP exposures as shown in Table 1. Figure 2 compares the HIP treatments according to their respective temperatures and cooling times. Variant 1 is denoted the as-built condition, where no HIP was applied. Variants 2 through 4 were HIPed under the same pressure of 103 MPa for 120 min. Variant 2 was held at a temperature of 850 °C, which is below the beta transus. Variant 3 was held at a standard HIP temperature of 950 °C, and finally, variant 4 was held at a temperature of 1050 °C, which is above the beta transus (~994 °C). For the three HIPed variants, a QIH9 system with Uniform Rapid Cooling (URC) furnace in molybdenum (Quintus Technologies LLC, Lewis Center, OH, USA) was used at a furnace cooling rate of 100 °C/min.

### 2.5. Specimen Test Preparation

Metallographic and mechanical test samples were fabricated by Honeywell Aerospace, in the two systems, an Arcam Q20 and A2X (Arcam AB, Mölnlycke, Sweden). Honeywell Aerospace has collaborated with Quintus Technologies developing new HIP and heat-treating cycles for Titanium aerospace materials. A total of 38 specimens with an average length of 10 cm were produced on each system, then separated into four different groups for classifying HIP variants in a vertical position as well as one separate group built horizontally to analyze its as-built condition.

### 2.6. Microstructure Characterization

Samples were cut, at the threaded section in the X, Y and Z planes. After sectioning, the samples were mounted with black phenolic powder in an ATM™ OPAL 460 mounting system (ATM Qness, Haan, Germany), then grounded until plane using a grinding pad of grit size 220, applying a force of 35 N and 300 RPM on an ATM™ SAPHIR 530 semi-automatic grinder and polisher (ATM Qness, Haan, Germany). Consecutively, they were polished on the same system using a diamond pad, applying a force of 25 N and 150 RPM for 5 min, using a 6 µm diamond suspension liquid. Finally, the samples were polished using a polishing pad and applying alkaline 0.2 µm fumed silica for 5 min, the parameters for the machine were set with a force of 25 N and 150 RPM.

The microstructure was revealed using a Modified Kroll’s Reagent solution consisting of 92 mL of distilled water (H₂O), 6 mL of nitric acid (HNO₃), and 2 mL of hydrofluoric acid (HF). The polished surface of specimens was etched with this solution by submerging the sample in it approximately for 5 s and, depending on the HIP variant, the time changed. For variants 1 and 2, a second step in the etching process was necessary to reveal the grain boundaries, following the Kroll’s reagent solution, submersion in Weck’s reagent (3 g NH4HF2 in 100 mL of distilled water) for 10 s, ceasing when bubbles started to appear. The microstructure of the materials was studied using an Olympus™ GX53 inverted optical microscope (Olympus Inc., Tokyo, Japan).

### 2.7. Density Measurements

The volume of Ti-6Al-2Sn-4Zr-2Mo was calculated using an AccuPyc II 1340 gas pycnometer (Micromeritics Instruments, Norcross, GA, USA). The mass values were measured using a Sartorius CP124S weight balance (Sartorius AG, Gotinga, Germany). For measurement values, each sample was weighted five times, with measurements taken after the balance was tared, and the reported values are the result of the average mass divided by the volume of each specimen.

### 2.8. Tensile Testing

Testing was conducted on an MTS Landmark servo-hydraulic system (MTS, Minneapolis, MN, USA) with a force capacity of 100 kN, equipped with threaded grips. To test the samples, they were machined and threaded according to the ASTM E8 standard [5] to fit the grips of the testing machine. For the axial strain measurement, an MTS 30 mm axial clip extensometer was installed with caution, aiming at the middle portion of the sample, predicting the failure location, and performed the test in all specimens. The displacement rate (or strain rate) for the machine was set at 0.15 mm/min. Testing was performed on six specimens for each variant and the results were averaged to assure the repeatability of results.

### 2.9. Hardness Testing

A Struers Duramin-A300 (Struers, Cleveland, OH, USA) was utilized to obtain hardness measurements in the Rockwell C (HRC) scale from the E-PBF manufactured components. Measurements were done for both top and bottom sections of the specimens, performing measurements on the X, Y and Z planes. The measurements used 5-s dwell time indentations with a load of 100 gf. A total of four evenly distributed indentations were made on the surfaces of every specimen, each with a separation of at least one millimeter.

### 2.10. Fracture Surface Characterization

After tensile testing, the fracture surface of one Ti 6Al-2Sn-4Zr-2Mo specimen per variant was examined using a JEOL JSM-IT500 SEM (JEOL, Tokyo, Japan). One end of the broken specimen was mounted to perform observations of its surface, as well as to compare fractures between the different systems.

### 2.11. Grain Size Measurements

Grain size measurements were performed according to ASTM E 112 standards test methods for determining grain size measurements [25] using the intercept procedure. Estimation of the average grain size by counting the number of grains intercepted by one or more straight lines that are sufficiently long to yield at least 50 intercepts.

## 3. Results and Discussion

### 3.1. Microstructures and Microstructure Analysis

**Figure 3:**
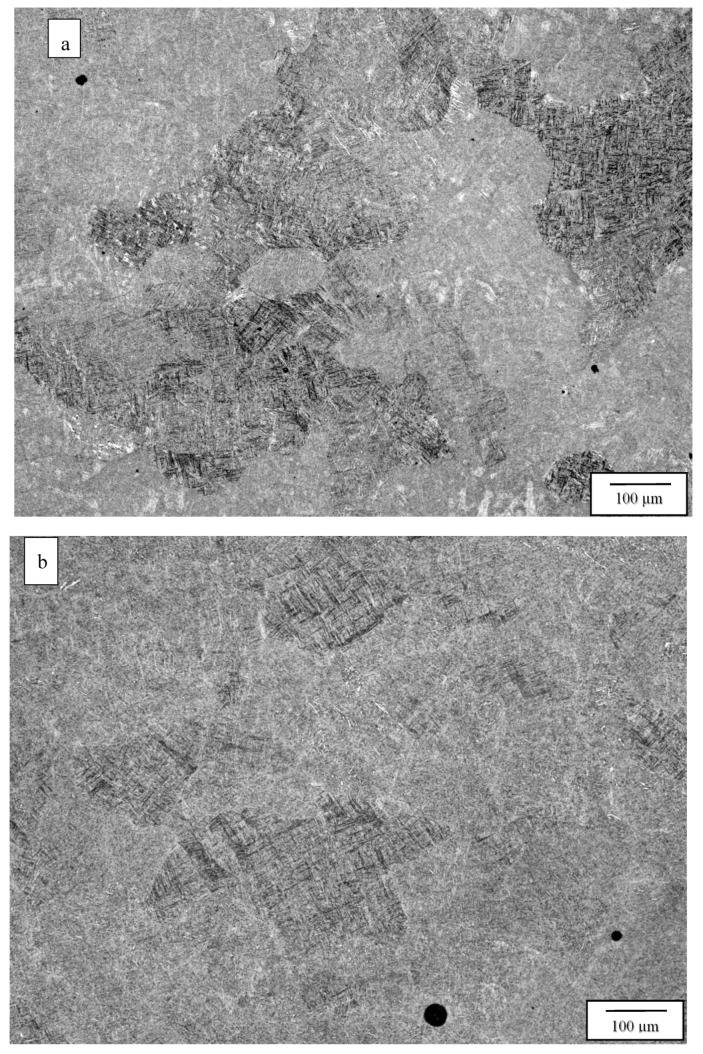
Variants 1 (as-fabricated) optical metallograph images for the Arcam A2X (**a**), and Q20 (**b**) top section, X plane.

**Figure 4:**
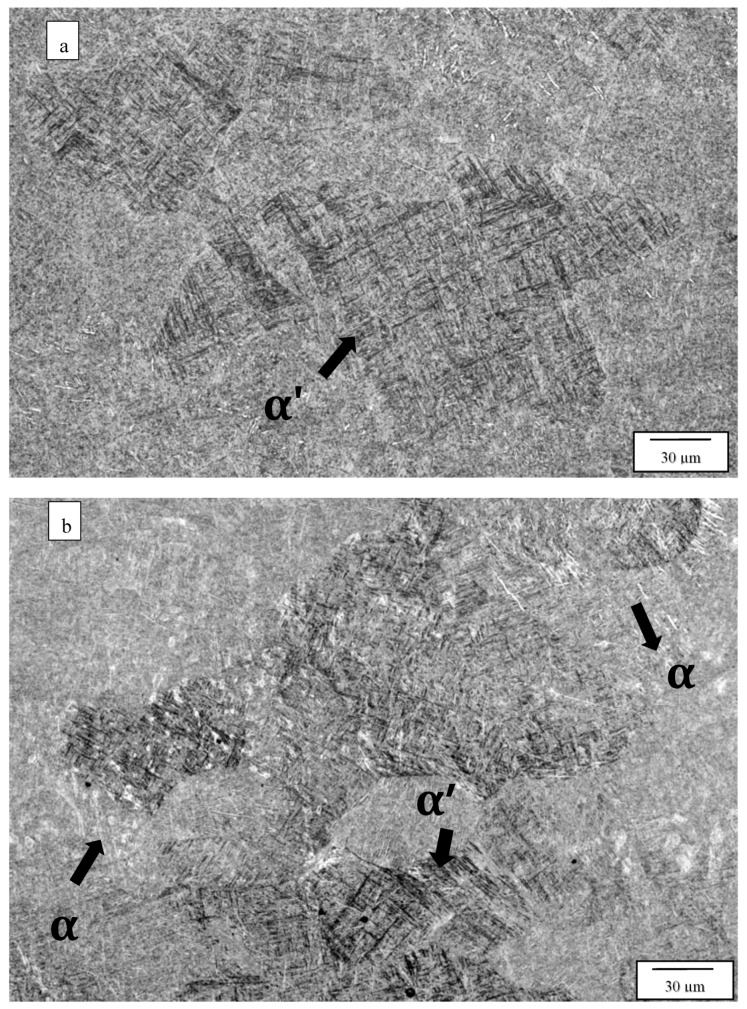
Variants 1 magnified images for the Arcam A2X (**a**), and Q20 (**b**) top section, X plane.

Figure 3 compares the as-fabricated (variant 1) microstructures for the Arcam A2X (Figure 3a) and Q20 (Figure 3b) EBM systems. Notable features are the equiaxed grain structures with similar grain sizes of 214 and 257 microns, respectively, for the A2X and Q20 EBM systems. These equiaxed grain structures correspond to moderate thermal gradients and solidification rates for solidification maps where plots of thermal gradient versus solidification rate determine the grain morphology differentiated by columnar grains versus varying sizes of equiaxed grains for both SLM and EBM fabrication of alloys, as recently reviewed by DebRoy, et al. [26]. Both images in Figure 3 show some porosity, and this is expected for measured densities of 4.51 and 4.49 g/cc, corresponding to Figure 3a,b, respectively. The nominal solid density for Ti6242 in the literature varies from ~4.52 to 4.54 g/cc [6,7], which compares favorably with the nominal, solid density of 4.53 g/cc measured for the specimens fabricated by both Arcam machines and HIPed at 1050 °C. Consequently, the measured densities for Figure 3a,b correspond to 99.6 and 99.2%, respectively. In Figure 3a,b the essentially fully dense, acicular alpha-prime martensite composing a Widmanstätten-like microstructure within the grains is also notable, having an average acicular thickness averaging ~0.6 microns. These features are better illustrated in the magnified images of Figure 3 shown in Figure 4. Since alpha-prime martensite has an hcp crystal structure and associated lattice parameters are essentially indistinguishable from the hcp alpha phase, it is difficult to distinguish from the alpha phase. However, in optical (light) metallography, the etchant renders the acicular alpha-prime dark or black as shown in the large grain marked in Figure 4a, while the acicular alpha phase appears white, as marked in Figure 4b. In addition, while acicular alpha occurs somewhat randomly, the alpha-prime martensite is often coincident with the hcp {0001} planes which can intersect at 90° as evident in many of the grains in Figure 4a,b. It can also be observed that some grains and grain portions contain short, acicular alpha with the same thickness as the martensite, as evidenced by the short, white features noted in Figure 4b. Nonetheless, the volume fraction of the acicular martensite (as observed qualitatively) far exceeds the acicular alpha. These features were observed more than three decades ago for the weld fusion zone of rapidly cooled Ti6242 alloy by Blaeslack and Mullins [20] where, at very high cooling rates (~500 °C/s), martensite dominated the microstructure, while an increasing volume fraction of acicular alpha emerged with decreasing cooling rates (by alpha-prime decomposition to alpha), creating alpha colonies at a cooling rate of ~7 °C/s. Similar microstructure features have been more recently observed in the laser-welding of both Ti6242 and Ti-6Al-4V (Ti64) alloys [21,27,28,29], and the same acicular alpha-prime martensite microstructures have been recently observed for the selective-laser melting (SLM) of Ti64 [11,12,30]. It is also of interest to note that Ter Haar and Becker [11] and Yang, et al. [13] have discussed a specific range of length regimes (from nano to micron) for alpha-prime martensite in Ti-6Al-4V, but their origin and specific influence on properties have not been discussed. In this regard, the short segments of alpha phase prominent in Figure 4b are also not understood.

**Figure 5:**
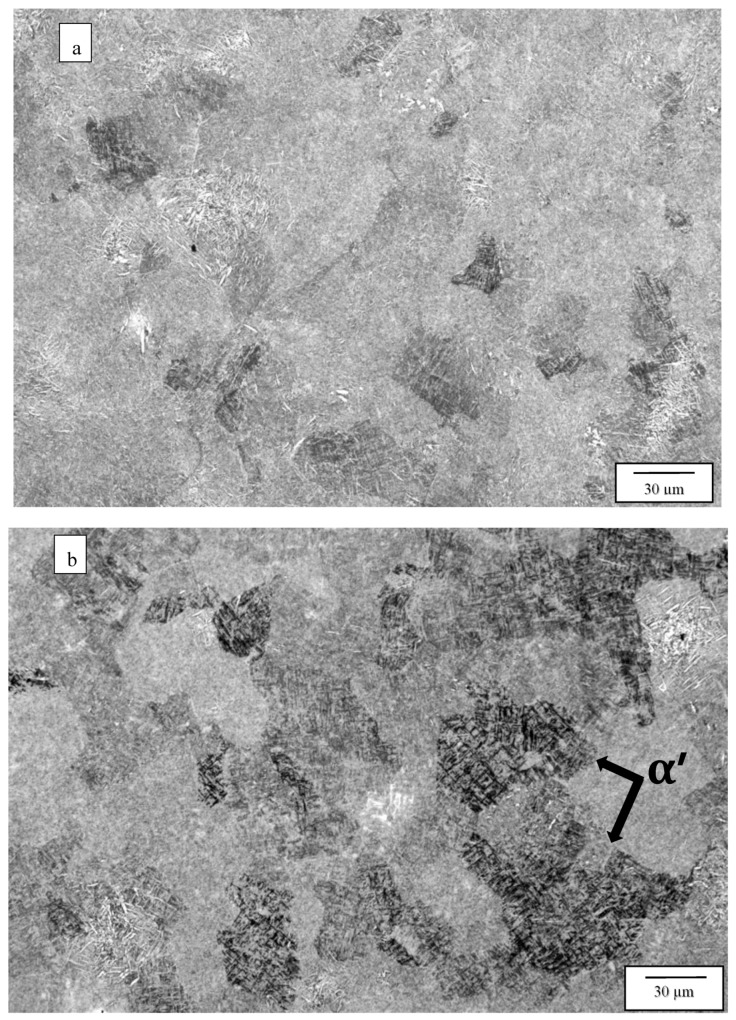
Variants 2 images for the Arcam A2X (**a**), and Q20 (**b**) top section, X plane.

Figure 5 compares the post-process HIP microstructures for the two Arcam EBM systems, at 850 °C (variant 2) as noted. It can be observed on comparing these microstructures with the as-fabricated (variant 1) microstructures in Figure 4 (at the same magnification) that the grain structure has been refined, leaving somewhat blocky grain sizes of ~121 microns and 165 microns for Figure 5a,b, respectively. While the martensite has partially decomposed, a significant volume fraction of the alpha-prime martensite persists, there is also a more significant acicular alpha volume fraction, and the thickness of both the acicular alpha-prime and the alpha is ~0.7 microns. The grain refinement is probably associated with the stress relief of dislocation microstructures, although these have not been specifically observed.

**Figure 6:**
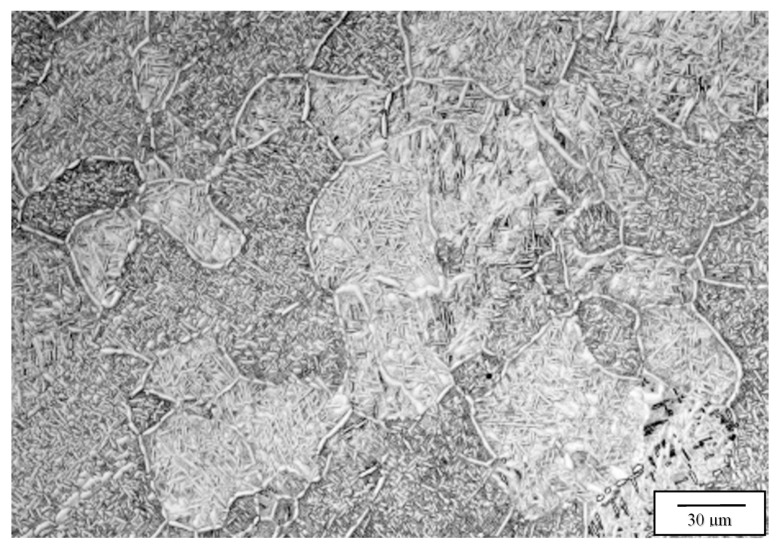
Variant 3 image for the Arcam Q20 top section, X plane.

**Figure 7:**
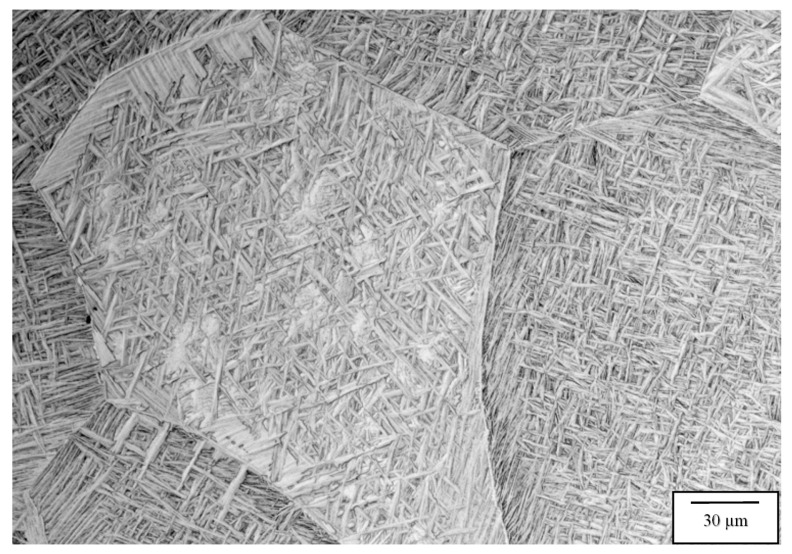
Variant 4 image for the Arcam Q20 top section, X plane.

**Figure 8:**
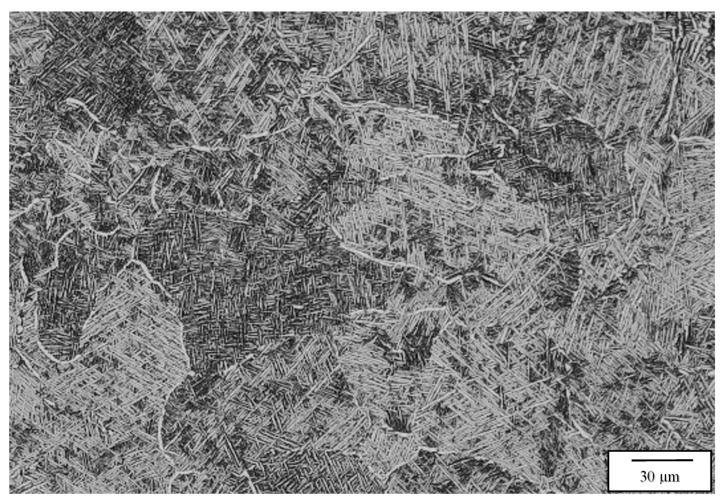
Variant 3 image for the Arcam A2X bottom section, X plane.

**Figure 9:**
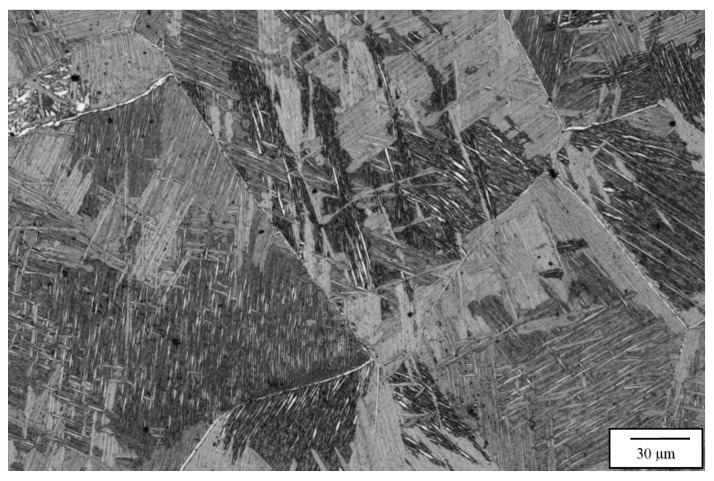
Variant 4 image for the Arcam A2X bottom section, X plane.

Figure 6, Figure 7, Figure 8 and Figure 9 compare the post-process, HIP microstructures at 950 °C (Figure 6 and Figure 7) and 1050 °C (Figure 8 and Figure 9), respectively. At 950 °C (variant 3), the equiaxed grains have grown slightly from the 850 °C HIP (Figure 5), but the most notable microstructure feature is the transformation of the acicular martensite to acicular alpha, represented by short segments of alpha in Figure 6 in contrast to a fairly well defined acicular alpha Widmanstätten microstructure in Figure 8. The acicular alpha in both Figure 6 and Figure 8 have an average thickness of ~0.6 microns. There is also a notable grain boundary regime of alpha in both Figure 6 and Figure 8. It might also be pointed out that the alpha phase in Figure 4b, Figure 6, Figure 7, Figure 8 and Figure 9 demonstrate a wide range of lamellar or acicular lengths, as noted above, from ~5 microns in Figure 4b and Figure 6, 20 microns in Figure 7 and 5 to 15 microns and ~5 to 60 microns in Figure 8 and Figure 9, respectively.

Figure 7 and Figure 9 compare the 1050 °C HIP (variant 4) post-process microstructures for the two different EBM machines. Here, the equiaxed grains have grown significantly in contrast to HIP at 850 and 950 °C HIP treatments shown in Figure 5, Figure 6 and Figure 8, respectively. In addition, the difference noted for the acicular alpha intra-grain microstructures is also shown when comparing Figure 7 and Figure 9, where a thick (~2 micron) acicular Widmanstätten microstructure occurs in Figure 7 in contrast to a thinner (~0.5 micron) acicular alpha pseudo- Widmanstätten microstructure in Figure 9. It should also be noted that the measured densities corresponding to the 1050 °C HIP (variant 4) corresponding to Figure 7 and Figure 9 were both 4.53 g/cc. Table 3 and Table 4 list the measured densities, grain sizes, and acicular alpha-prime and alpha thicknesses for comparison and reference, along with the corresponding mechanical properties.

### 3.2. Mechanical Property Measurements and Discussion

For comparison, Table 3 and Table 4 list and summarize the averages for the tensile and Rockwell C (HRC) hardness measurements and microstructure data for the four variants: as-processed (1), HIP at 850 °C (2), HIP at 950 °C (3), and HIP at 1050 °C (4), for the two Arcam EBM fabrication systems (Q20 and A2X, respectively). The stress at fracture is also listed. As expected, the tensile yield stress and UTS decrease regularly with HIP post-processing (increasing temperatures) for variants 2, 3, and 4, but with some anomalies in both fracture stress and associated elongation (ductility) as a consequence of the interactive microstructures: grain size changes (increase) along with variations in the acicular alpha-prime/alpha volume fractions. Even in the very early observations of Ti6242 weld fusion zone microstructures [13], the rich (fully dense) acicular martensite was associated with the highest tensile yield stress and UTS and the lowest elongation, while the yield stress and UTS declined regularly with increasing acicular alpha, and increasing elongation.

The hardness measured in this prior research [20] also decreased systematically by ~12%. In the present study, corresponding hardness also decreased somewhat inconsistently, as shown in Table 3 and Table 4, and only varied by ~7%. Similar results for tensile property variation with acicular alpha-prime/alpha content have also been observed for both variously welded Ti-6Al-4V [28,29], as well as laser (SLM) and electron beam (EBM) processing and heat treatment of Ti64 [7,9,10], where additional anomalies and superior ductility have been achieved by the post-heat-treatment processing.

It is apparent upon examining and comparing Table 3 and Table 4, along with a somewhat systematic perusal of the comparative microstructure images in Figure 4, Figure 5, Figure 6, Figure 7, Figure 8 and Figure 9, that there is a complex interplay between the grain structure (grain size) and the intragrain microstructures: acicular alpha-prime and alpha, including the contributions of bcc beta phase. Of course, it is well established that tensile yield stress is proportional to the reciprocal square root of the grain diameter. Consequently, as the grain size increases, as shown for the HIP variants (2, 3 and 4) in Table 3 and Table 4, the tensile yield would nominally decrease. Similarly, Xu, et al. [27] have shown that, for the Ti64 alloy, the yield stress is also inversely proportional to the square root of the acicular and lenticular alpha phase thickness, and for thicknesses ranging from ~3 microns to 0.3 microns, the yield stress increased from ~0.85 GPa to 1.1 GPa; a difference of ~29%. In this regard, it can be observed in Table 3 and Table 4 that, although the grain size (diameter) increases with HIP temperature, the acicular alpha thickness does not change significantly, except for the 1050 °C HIP treatment for the Q20 EBM-processed alloy where the alpha coarsened to a thickness of ~2 microns, and the tensile elongation decreased to 9.2%. There is also a simple relationship between tensile stress and hardness for many metals and alloys (tensile stress ~hardness/3), especially for Vickers and Rockwell C hardness, and this is also not a characteristic of the results presented in Table 3 and Table 4. However, since the hardness measurements represent intragrain regions characterized by acicular alpha-prime or alpha, which do not change significantly in thickness, the hardness is also not characteristically different, and remains relatively high in comparison with the hardness for annealed Ti6242 alloy, which is near HRC 33 [6,7].

Even though there are anomalies in correlating the Ti6242 EBM-processed and post-processing HIP microstructures (Figure 4, Figure 5, Figure 6, Figure 7, Figure 8 and Figure 9) with the corresponding mechanical properties, the results show clearly defined microstructures for the various Ti6242 specimens, and allow useful conclusions to be drawn regarding the effects of post-processing HIP conditions on selecting and optimizing an EBM-fabricated Ti6242 alloy. For example, Table 3 and Table 4 illustrate that, for the low temperature (850 °C) HIP, the Ti6242 has a density equal to or greater than 99.6%, while the elongation (ductility) is as good or better than the same properties for conventionally processed wrought alloy [6,7]. Another interesting feature is that, for some Titanium alloys, the elongation has been shown to be inversely proportional to the volume fraction of acicular alpha [31], and this may contribute to the decline in elongation observed for variants 3 and 4 in Table 3 and Table 4 relatives to variant 2. The creep rupture resistance also normally declines with a decline in elongation, although this feature was not directly measured in this study.

### 3.3. Tensile Fracture Surface Observations and Analysis

**Figure 10:**
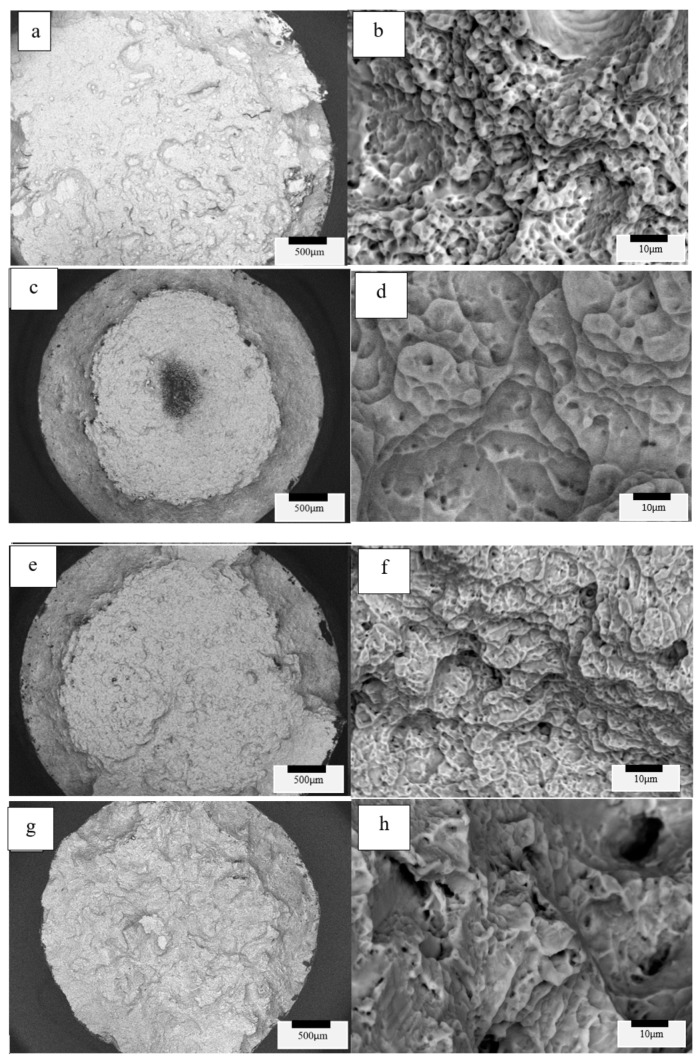
Variants 1 (**a**,**b**), 2 (**c**,**d**), 3 (**e**,**f**), 4 (**g**,**h**) for the Arcam A2X-processed and HIP post-processed Ti6242 tensile fracture surfaces.

**Figure 11:**
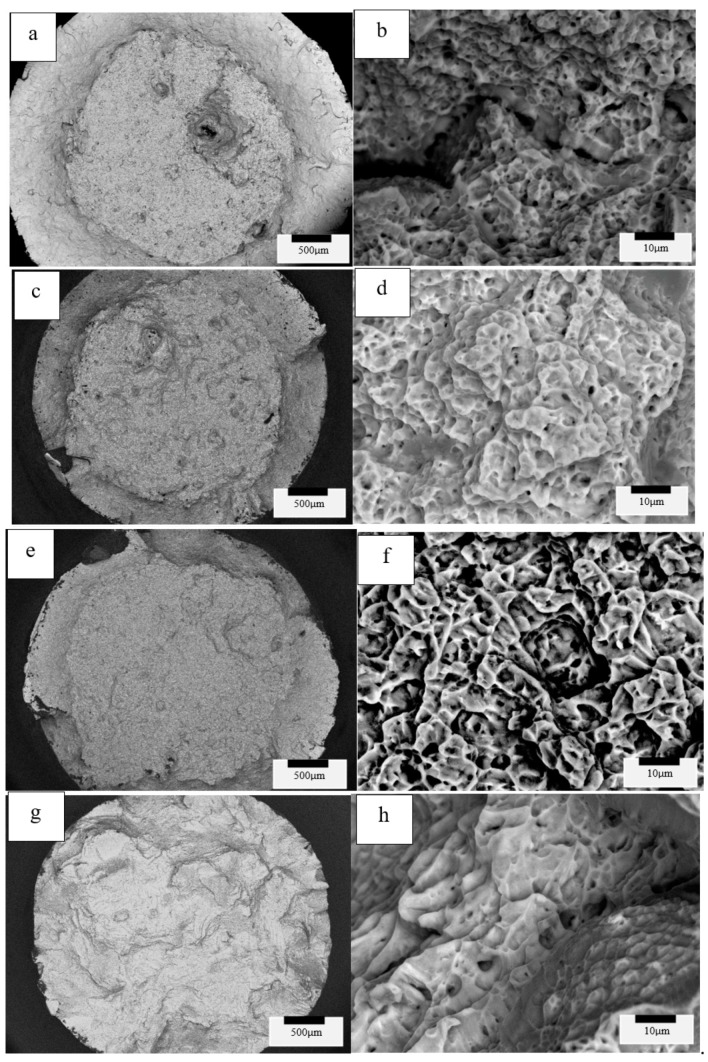
Variants 1 (**a**,**b**), 2 (**c**,**d**), 3 (**e**,**f**), 4 (**g**,**h**) for the Arcam Q20-processed and post-processed tensile fracture surfaces.

SEM fractographic images for the four variant test categories (variants 1, 2, 3 and 4) implicit in Table 3 and Table 4 are shown for comparison in Figure 10 and Figure 11, as low- and high-magnification sequences. Evidence for ductile dimple fracture features is observed in all of the high-magnification images to the right in Figure 10 and Figure 11, where the average dimple diameters have been estimated to be 2.5, 5, 2 and 8 microns, respectively, for the four variants top to bottom at the right in Figure 10, and 2.5, 4, 5 and 7 microns, respectively, for the four variants top to bottom at the right in Figure 11; generally increasing with increasing grain size. This behavior has been described in earlier work on titanium by Krishna et al. [32], and more recently for a Titanium alloy by Illarionov et al. [33]. In addition, deeper dimples also generally indicate greater toughness, although toughness was not specifically measured in this study. Dimples are also shallower as the grain size (diameter) increases, and this is observed in both Figure 10h and Figure 11h, corresponding to HIP at 1050 °C (variant 4), where the grain size is largest (Table 3 and Table 4).

## 4. Summary and Conclusions

The microstructure for the EBM-fabricated Ti6242 consists of equiaxed grains (~200–300 microns in diameter) containing a nearly total volume fraction of acicular alpha-prime martensite having a width of ~0.6 microns. These results were essentially unchanged for samples fabricated in two different Arcam EBM systems: A2X employing a tungsten thermionic filament electron source, and Q20 employing a LaB_6_ thermionic electron source. The processing parameters for each machine (Table 1 and Table 2) produced similar thermal gradients and solidification rates and, ultimately, high cooling rates.

Post-process HIP of the as-fabricated Ti6242 components at temperatures of 850 °C, 950 °C and 1050 °C produced blocky, refined grains containing a mixture of acicular martensite and alpha at 850 °C, and increasing grain growth along with the decomposition of intragranular, acicular alpha-prime martensite to produce thin, acicular, and Widmanstätten alpha phase at the two higher temperatures. The corresponding tensile properties (yield and UTS) declined from ~1 and 1.1 GPa, respectively, to ~0.8 GPa and 0.9 GPa, respectively for HIP processing at 1050 °C for both Arcam EBM systems where the corresponding hardness varied from 41 to 38 HRC. While there were some anomalies in the elongation (ductility), the optimized, post-process HIP was associated with 850 °C where the elongation varied between 15 and 16%, corresponding to a yield strength of 0.9 GPa and UTS of ~1 GPa. Of course, this optimized, post-process HIP applies only to the very preliminary treatments in this study.

## Figures and Tables

**Table 1:** Build Parameters for Arcam A2X specimens.

Machine Build Parameters Arcam A2X			
Pre Heat 50 µm	Pre Heat	Focus offset	62 mA
		Heating Focus offset	130 mA
		Offset to part	4 mm
	Pre Heat 1	Max Beam Current	30 mA
		Beam Speed	1300 mm/s
		Max No. of repetitions	25
		Average current	10 mA
	Pre Heat 2	Max Beam Current	38
		Beam Speed	14,600 mm/s
		Max No. of repetitions	12
		Average current	12.2 mA
		Max Heat time	15–20 s
Melt 50 µm	Outer Contour	No. of spots	40 ms
		Spot time	0.8 mm
		Multispot Overlap	0.2 mA
		Current	4 mA
		Focus offset	3
		Speed Function	6
	Inner Contour	Current	10
		Focus offset	3
		Speed Function	30
	Hatch	Current	15 mA
		Focus offset	3
		Speed Function	98
		Line Order	1
	Heating	Max heat time	55 s
		Layer thickness	0.05 µm

**Table 2:** Build Parameters for Arcam Q20 specimens.

Machine Build Parameters Arcam Q20			
Pre Heat 50 µm	Pre Heat	Focus offset	46 mA
		Heating Focus offset	100 mA
		Offset to part	2 mm
	Pre Heat 1	Max Beam Current	36 mA
		Beam Speed	42,000 mm/s
		Max No. of repetitions	2
		Average current	N/A
	Pre Heat 2	Max Beam Current	38
		Beam Speed	40,500 mm/s
		Max No. of repetitions	3
		Average current	12.25 mA
		Max Heat time	N/A
Melt 50 µm	Outer Contour	No. of spots	N/A
		Spot time	N/A
		Multispot Overlap	N/A
		Current	5 mA
		Focus offset	7
		Speed Function	N/A
	Inner Contour	Current	8
		Focus offset	7
		Speed Function	N/A
	Hatch	Current	15 mA
		Focus offset	38
		Speed Function	60
		Line Order	N/A
	Heating	Max heat time	30 s
		Layer thickness	0.05 µm

**Table 3:** Mechanical Properties of Ti 6Al-2Sn-4Zr-2Mo specimen variants built on an ARCAM Q20 system.

Ti 6Al-2Sn-4Zr-2Mo Properties Arcam Q20								
Variant	Avg. σy	Avg. UTS	Avg. Fracture	Avg. Ɛmax	Avg. Density	Avg. Hardness	Grain Size	Alpha-Prime/Alpha
	(MPa)	(MPa)	(MPa)	Elong.	(g/cm³)	(HRC)	(µm)	Thickness (microns)
1	957 (±16)	1059 (±19)	979 (±64)	13.8% (±0.02%)	4.49 (±0.2)	39 (±0.4)	257	0.6
2	896 (±21)	1013 (±29)	830 (±47)	15.3% (±0.01%)	4.51 (±0.2)	38 (±0.4)	165	0.7
3	862 (±46)	963 (±7)	822 (±42)	12.3% (±0.03%)	4.52 (±0.2)	38 (±0.9)	217	0.6
4	832 (±14)	935 (±19)	896 (±18)	9.2% (±0.02%)	4.53 (±0.2)	38 (±0.3)	347	2.0

**Table 4:** Mechanical Properties of Ti 6Al-2Sn-4Zr-2Mo specimen variants built on an ARCAM A2X system.

Ti 6Al-2Sn-4Zr-2Mo Properties A2X								
Variant	Avg. σy	Avg. UTS	Avg. Fracture	Avg. Ɛmax	Avg. Density	Avg. Hardness	Grain Size	Alpha-Prime/Alpha
	(MPa)	(MPa)	(MPa)	Elong.	(g/cm³)	(HRC)	(µm)	Thickness (microns)
1	1018 (±14)	1115 (±18)	1074 (±17)	7.8% (±0.03%)	4.51 (±0.2)	41 (±0.3)	214	0.6
2	937 (±19)	1048 (±7)	824 (±46)	16.4% (±0.03%)	4.52 (±0.2)	39 (±1)	121	0.7
3	887 (±21)	1000 (±4)	840 (±60)	16.2% (±0.01%)	4.52 (±0.2)	38 (±0.5)	278	0.6
4	836 (±8)	963 (±6)	874 (±41)	12.2% (±0.02%)	4.53 (±0.2)	41 (±1)	329	0.5
